# Enhanced recovery after surgery-guided strategies in single-incision laparoscopic totally extraperitoneal hernioplasty for inguinal hernia: a narrative review

**DOI:** 10.3389/fsurg.2025.1626784

**Published:** 2025-07-22

**Authors:** Yi Jin, Hongqun Zhang, Yuping Chen, Chunyan Zhang, Yan Lin, Zhuoyin Wang

**Affiliations:** General Surgery, Ningbo Beilun Third People’s Hospital, Ningbo, China

**Keywords:** SIL-TEP, single-incision laparoscopic, inguinal hernia, ERAS, postoperative care, treatment, improved recovery rate

## Abstract

**Objective:**

To investigate the clinical efficacy and implementation challenges of single-port laparoscopic total extraperitoneal hernia repair (SIL-TEP) combined with enhanced recovery after surgery (ERAS) in the treatment of inguinal hernia.

**Methods:**

This review summarized the technical advantages of SIL-TEP in reducing postoperative pain, accelerating functional recovery and improving cosmetic results compared with traditional three-port TEP. The perioperative strategies under eras concept were further discussed, including preoperative nutrition optimization, laryngeal mask airway (LMA) use, early oral feeding, multimodal analgesia and timely removal of urinary catheter.

**Results:**

SIL-TEP combined with ERAS had significant clinical benefits, including decreased pain score at 24 h after operation, shortened recovery time of daily activities, and improved patient satisfaction with incision appearance. ERAS interventions have resulted in reduced length of hospital stay; however, there are still technical limitations, including difficulties in device triangulation and learning curve requirements for the number of medical records.

**Conclusions:**

The collaborative application of SIL-TEP and ERAS represents a paradigm shift in minimally invasive hernia management, achieving enhanced recovery metrics and cost-effectiveness. Although the current evidence supports superiority in the short term, more multicenter randomized trials (RCTS) with 5-year follow-up are needed to verify long-term recurrence rates and socioeconomic impact. Standardized training programs and AI-assisted surgical systems may address existing technical barriers to widespread adoption.

## 1 Introduction

Inguinal hernia is one of the most common types of hernias encountered in clinical practice (1). Patients may present with a bulge in the inguinal region that gradually enlarges over time. While most patients report pain or discomfort, approximately one-third remain asymptomatic (2). Symptoms can worsen during activities such as standing, straining, lifting heavy objects, or coughing, as these actions increase intra-abdominal pressure and cause abdominal contents to protrude through the defect (3). In some patients, the bulge may disappear when lying supine. In certain cases, groin or pelvic pain is caused by occult hernias (also termed hidden hernias) (4). The HerniaSurge guidelines recommend that physicians educate asymptomatic or minimally symptomatic patients with inguinal hernias about the natural progression of the disease and the risks associated with emergency surgery (5). However, patients often express concerns about surgical risks, postoperative recovery, and hospitalization costs. Therefore, implementing Enhanced Recovery After Surgery (ERAS) protocols is crucial for shortening treatment duration, reducing hospitalization expenses, alleviating postoperative pain, and improving patient satisfaction.

Surgical intervention remains the gold standard for the treatment of inguinal hernias (6). Current approaches include open repair, tension-free mesh repair, and laparoscopic techniques—the latter comprising transabdominal preperitoneal (TAPP) and totally extraperitoneal (TEP) methods, both of which now incorporate single-incision laparoscopic approaches (SIL-TAPP and SIL-TEP) (5, 7). Evidence from comparative studies demonstrates that laparoscopic repair achieves superior postoperative pain outcomes compared to tension-free mesh repair (5). When contrasted with traditional open tension-free techniques, laparoscopic surgery offers distinct advantages, including smaller incisions, reduced postoperative pain, and accelerated return to normal activities (8–11). Notably, This meta—analysis demonstrates that TEP and TAPP have comparable rates of hernia recurrence and postoperative chronic pain. The trial sequential analysis indicates that the information size is sufficient. Future trials are unlikely to reveal a significant difference between the two techniques and should generally be avoided (12). The recent European Hernia Society's guidelines indicate that Lichtenstein tension—free and minimally invasive techniques (e.g., TAPP and TEP), when performed by expert surgeons, are recommended as the optimal evidence—based options for inguinal hernia repair (5). However, a recent network analysis of RCTs shows that both TEP and TAPP appear to be associated with a reduced risk of postoperative pain and a shorter return to work and daily activities compared to open tension—free repair (13). Key advantages of TEP include avoidance of intraperitoneal entry, elimination of peritoneal closure requirements, and enhanced postoperative recovery timelines. Emerging data suggest that patients undergoing SIL-TEP achieve comparable clinical outcomes to those treated with conventional TEP, with the exception of recovery metrics. Specifically, studies indicate that the SIL-TEP group exhibits shorter recovery periods and significantly lower short-term pain scores (Visual Analog Scale, VAS) (14, 15).

The concept of ERAS was first introduced in the 1990s (16). This evidence-based paradigm optimizes clinical pathways by minimizing surgical stress, alleviating pain, shortening hospital stays, and accelerating postoperative recovery (17–20). In recent years, the ERAS framework has progressively expanded, with evolving perioperative care protocols incorporating novel interventions (21). At our institution, a dedicated registered nurse (RN) is assigned to each patient upon admission to coordinate all ERAS-related activities. The RN's role encompasses perioperative counseling to address patient inquiries and reduce anxiety, coupled with comprehensive management of preoperative protocols (e.g., skin antisepsis and dietary optimization), intraoperative measures (e.g., normothermia maintenance, refined anesthesia strategies, and catheter management), and postoperative interventions (e.g., multimodal analgesia and early ambulation). This article highlights our latest advancements in integrating ERAS protocols into SIL-TEP procedures, demonstrating how structured multidisciplinary collaboration enhances both clinical outcomes and patient-centered care.

## 2 Application of SIL-TEP in inguinal hernia surgery

### 2.1 Evolution of SIL-TEP: From concept to clinical practice

The minimally invasive revolution in inguinal hernia repair has driven iterative refinements beyond traditional open anterior approaches. Following the maturation of TEP, surgical innovation focused on further minimizing operative trauma through port reduction strategies. This technological convergence led to the adaptation of single-incision laparoscopic surgery (SILS) for inguinal hernia management. In 2008, Cugura et al. pioneered SIL-TEP, marking its formal entry into clinical practice (7). Subsequent decade-long investigations have systematically evaluated SIL-TEP's technical feasibility and safety profile. Current evidence demonstrates that SIL-TEP achieves comparable recurrence rates to conventional TEP when performed by experienced surgeons, while offering potential advantages in the minimally invasive nature of incisions, enhanced postoperative pain control, and accelerated functional recovery. These outcomes position SIL-TEP as a promising frontier in minimally invasive hernia repair, prompting ongoing research into its standardization and broader clinical adoption (14, 15, 22, 23, 24).

### 2.2 Patient selection and contraindications

Ideal candidates for SIL-TEP include primary unilateral inguinal hernia patients with BMI <30 kg/m^2^. Contraindications include: Previous lower abdominal surgery (risk of adhesions), Irreducible/incarcerated hernias (relative contraindication), Severe cardiopulmonary disease (inability to tolerate pneumoperitoneum), Complex bilateral or recurrent hernias (surgeon-dependent) (5).

### 2.3 SLE-TEP surgical technique

Combined intravenous and inhalational general anesthesia was used for surgery, and indwelling catheter was used before surgery. The skin and subcutaneous tissue were incised with an arc-shaped dermoid incision of 2–2.2 cm through the lower edge of the umbilicus, and the abdominal linea alba was transected about 5–10 mm below the periumbilical fascia. For patients with narrow linea alba, bilateral rectus abdominis sheath should be incised, with a total length of about 2 cm. A preperitoneal space was then bluntly and sharply separated under direct vision at the level behind the posterior sheath posterior, and a single-Port was inserted. After the installation of the single-Port device, the surgical channel was separated between the posterior rectus sheath and the preperitoneum under direct vision with an electric hook and noninvasive forceps, where the Retizus space could be entered from the level in front of the transversalis fascia. It then expands to both sides into the Bogros space. After the Bogros space was initially established and the lateral peritoneal return line was separated, the hernia sac on one side was treated first. The hernia sac of direct hernia and minor indirect hernia should be completely stripped as far as possible. For large scrotal hernia and recurrent indirect hernia, active incision of the hernia sac and transection of the distal inner ring of the hernia are mostly used, and then the needle is pulled straight with 3-0 barbed suture and sutured continuously. The contralateral hernia was then treated. The methods were as described previously. The pre-cut single mesh was inserted after the separation was completed. The specific mesh cutting method was used to design the mesh according to the size of the hernia ring. The diameter of the direct hernia ring is greater than 3 cm, and the height is 12 cm. For cases less than 2.5 cm, the height was 10.5 cm. The width of the mesh is the distance between the patient's anterior superior iliac spine and the pubic symphysis. The measured data from each case are shown in Table 1, and the distance is between 25 and 30 cm. In practice, 26–30 cm is used. The mesh was flattened and deflated, and the incision was sutured.

**Table 1:** Pelvic anatomical measurements for mesh sizing reference in inguinal hernia repair.

Measurement type	Umbilicus to ASIS	Umbilicus to pubic symphysis	Inter-ASIS distance	ASIS to pubic symphysis
Mean ± SD (cm)	14.22 ± 1.13	14.05 ± 1.24	24.96 ± 2.10	14.05 ± 1.04

### 2.4 Application for SIL-TEP

As an innovative application of single-incision laparoscopic techniques, the SIL-TEP completes the extraperitoneal procedure through a single periumbilical incision. The core of this technique lies in overcoming the challenge of instrument crossover interference within a confined space. Although this surgical approach demands higher levels of spatial perception and bimanual coordination from surgeons, clinical practice has demonstrated its applicability to complex cases, including irreducible and incarcerated hernias (25). Cugura's landmark study not only established the standard operating procedures for SIL-TEP but also validated its safety through long-term follow-up data. Notably, the concealed umbilical incision of SIL-TEP significantly enhances cosmetic outcomes (14). Moreover, its high compatibility with the ERAS concept—including reduced accelerated postoperative functional recovery—positions it as an important direction in modern hernia surgery research. In the future, we will explore the use of dedicated single-port systems and articulating instruments to reduce the complexity of the surgical procedure, thereby opening up new avenues for the broader adoption and promotion of this technique.

### 2.5 Advantages and disadvantages of SIL-TEP

Advantages: SIL-TEP is a minimally invasive surgical technique that offers superior cosmetic results compared to traditional laparoscopic TEP (24, 26) (Figuress 1, 2). There are no significant differences between SIL-TEP and conventional TEP in terms of blood loss, complications, or recurrence rates. However, SIL-TEP is associated with less postoperative pain (30% reduction in postoperative pain [Visual Analog Scale (VAS) scores], shorter treatment cycles, reduced hospitalization costs, decreased abdominal trauma, alleviated postoperative discomfort, and improved patient satisfaction (27, 28).

**Figure 1:**
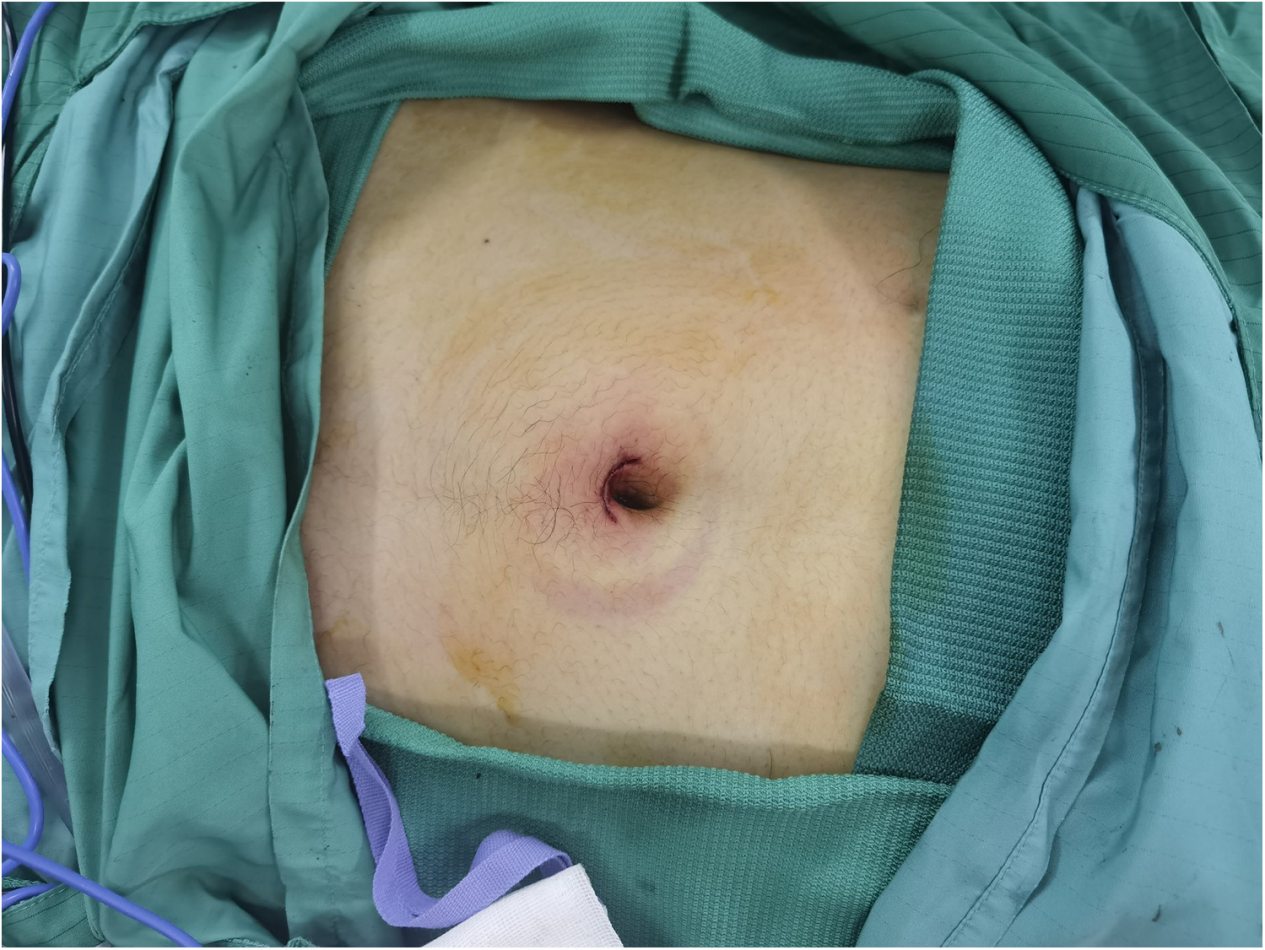
Umbilical incision at completion of surgery.

**Figure 2:**
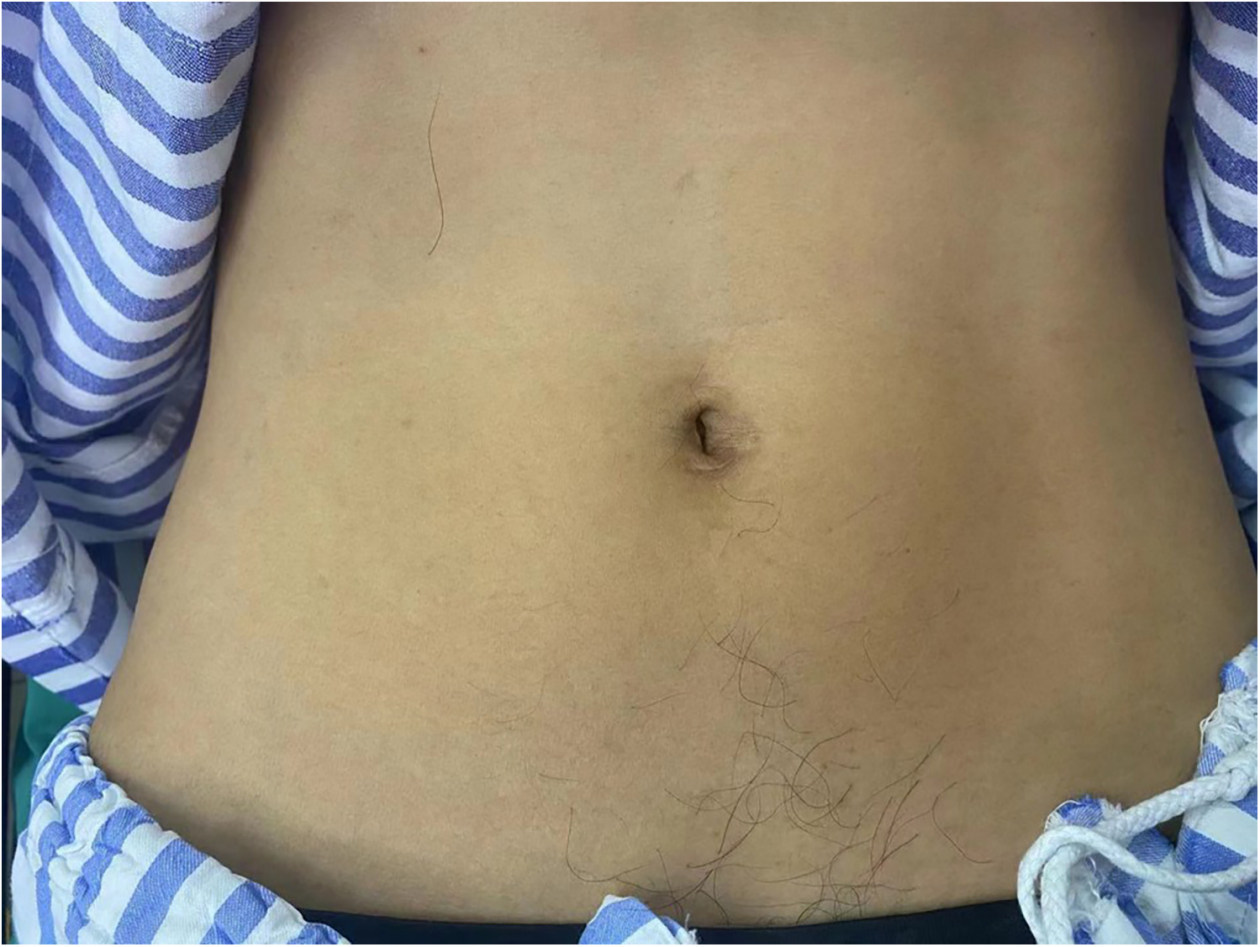
Umbilical incision one month after surgery.

Disadvantages: The initial experience with this novel surgical procedure can be time-consuming. This technique requires higher levels of spatial perception and bimanual coordination from the surgeon and necessitates managing instrument crossover challenges within a confined operative space. Considering the learning curve, it is expected that operative times will decrease as experience increases (29, 30).

### 2.6 Challenges and prospects in the application of SIL-TEP

As technology advances, specific problems and limitations have become apparent. For example, some contend that the limited operative space, instrument interference, increased surgical difficulty, and steep learning curve may heighten the risk of complications (31). Recent developments have focused on addressing these issues, such as exploring the use of specialized single-port systems and articulating instruments to reduce operative complexity. These innovations provide new perspectives for the promotion and adoption of SIL-TEP in clinical practice.

## 3 ERAS concept and development

### 3.1 Concept of ERAS

ERAS refers to the use of evidence-based, multidisciplinary treatment strategies designed to optimize various perioperative medical practices and nursing measures. The goal is to reduce surgical stress, minimize complications, and accelerate patient recovery (21, 32, 33). The core principle of ERAS is to implement the best evidence-based perioperative care measures to achieve rapid recovery. The ERAS concept encompasses four main components: (1) Multidisciplinary Collaboration: Integrating the expertise of various professionals. (2) A Multimodal, Problem-Solving Approach: Addressing the challenges of the perioperative period from multiple angles. (3) Scientific and Evidence-Based Care Planning: Developing individualized care plans based on validated protocols. (4) Interactive and Continuous Auditing in Management: Employing regular reviews and feedback to refine practices (34).

### 3.2 Development of ERAS

Fast Track Surgery (FTS) was initially proposed in the 1990s with the objective of facilitating rapid and safe patient discharge. As medical knowledge evolved, FTS was gradually replaced by ERAS, which places greater emphasis on the quality of rapid patient recovery. In 1997, Professor Kehlet from Denmark systematically introduced the ERAS protocol by demonstrating that multimodal interventions can effectively reduce the postoperative stress response (35). Following the release of the first international guidelines in 2012, the application of ERAS expanded from gastrointestinal surgery to 12 other fields, including orthopedics and urology, achieving an average reduction in hospital stay by 60% (36). Over the years, the ERAS protocol has gained widespread recognition, greatly emphasizing the collaborative efforts of multidisciplinary teams—comprising dietitians, surgeons, anesthesiologists, and nurses—to enhance overall surgical care quality across the preoperative, intraoperative, and postoperative phases. This multimodal approach has been proven to shorten hospitalization, alleviate postoperative pain, improve patient satisfaction, and expedite recovery (18). Current developments focus on AI-assisted decision making, and the emergence of Artificial Intelligence (AI) along with Machine Learning (ML) offers a promising avenue for further optimizing ERAS protocols (37).

## 4 Application of ERAS in the perioperative care of patients undergoing SIL-TEP inguinal hernioplasty

In recent years, our experience has shown that the implementation of ERAS protocols in SIL-TEP inguinal hernioplasty can markedly shorten treatment duration, reduce hospitalization costs, alleviate postoperative pain, and improve patient satisfaction. Consequently, the application of ERAS principles in SIL-TEP procedures is garnering increasing attention. However, evidence regarding the long-term benefits of ERAS in SIL-TEP patients remains scarce; Current results do not decisively demonstrate sustained improvements in patient outcomes. We therefore anticipate that further studies in the coming years will provide additional clarity.

### 4.1 Preoperative care

#### 4.1.1 Psychological care and health education

As healthcare delivery models evolve, patient care demands have become increasingly sophisticated. This has made the exploration of novel care models a prominent research topic in the medical field. Literature and clinical data indicate that nursing approaches emphasizing psychological care and health education are particularly beneficial. When informed about the impending surgery, patients with inguinal hernia often experience considerable psychological stress and emotional fluctuations, which can erode their confidence in the treatment. Concerns regarding surgical risks and postoperative recovery may lead to anxiety that adversely affects both the therapeutic and nursing outcomes. In such a context, psychological interventions integrated into the ERAS protocol are vital to enhancing patient compliance (38). With a patient-centered approach, the ERAS program seeks to empower patients at every care stage; improved education about their condition and treatment contributes to better adherence, reduced anxiety, and higher satisfaction (39, 40). RN provides overall coordination and patient-centered psychological care throughout the process. This is the core of ERAS, especially in addressing the patients' concerns that may arise from new technologies (SIL-TEP).

#### 4.1.2 Preoperative skin preparation

In single-incision laparoscopic procedures, the periumbilical incision is favored due to its ergonomic alignment with anatomical landmarks and the resultant optimization of operating triangulation. Given the unique microenvironment of skin folds in the umbilical region, meticulous preoperative cleansing is imperative. The umbilical fossa, prone to dirt and contaminants accumulation over decades, can't be thoroughly cleansed by routine preoperative povidone-iodine disinfection. Single-incision procedures without ERAS protocols also don't focus on preoperative skin preparation. However, our protocol combining liquid paraffin and povidone-iodine offers a comprehensive preoperative skin preparation approach. Our protocol begins with the application of liquid paraffin to dissolve and mobilize accumulated debris within the umbilicus. This is then followed by the localized application of povidone-iodine to the surgical area, effectively removing residual contaminants. It ensures thorough decontamination, effectively reducing postoperative incision—related complications like infection and fat liquefaction.

Liquid paraffin is a colorless, transparent oil—odorless, tasteless, and possessing a neutral acidic pH—that minimizes skin irritation. Its excellent lubricating properties render it a preferred agent in various invasive procedures. Based on our clinical experience, liquid paraffin works swiftly and effectively to soften and remove debris, thereby reducing the discomfort associated with repetitive cleaning and enhancing overall nursing efficiency (41).

Povidone-iodine, which consists of an iodine carrier that reacts with oxygen-containing functional groups and hydroxyl groups that denature bacterial proteins, targets nucleotides, sulfhydryl groups, and fatty acids. Through oxidative mechanisms and suppression of microbial protein synthesis, it effectively eradicates pathogens. According to the U.S. Centers for Disease Control and Prevention (CDC) guidelines for the prevention of surgical site infections, both chlorhexidine and povidone-iodine are appropriate for preoperative skin disinfection, significantly lowering the risk of surgical site infections.

The synergistic use of liquid paraffin and povidone-iodine establishes a comprehensive preoperative skin preparation protocol that not only ensures thorough decontamination but also plays a pivotal role in preventing postoperative wound infections, thereby enhancing patient safety during the perioperative period, and is an innovative, optimized nursing intervention for SIL-TEP.

#### 4.1.3 Dietary management

Traditional surgical approaches may induce gastrointestinal discomfort, potentially affecting surgical outcomes, exacerbating clinical distress, and, in severe cases, leading to complications. Accordingly, strict dietary control is imperative prior to surgery. We recommend that patients undergo an 8-h fasting period before surgery, with the provision of a small volume of liquid glucose approximately 2 h preoperatively in order to mitigate postoperative hunger (42). This protocol minimizes the risk of aspiration during the operation, prevents thirst and hypoglycemia in the perioperative phase, facilitates smooth conduct of the procedure, and reduces patient discomfort related to prolonged fasting and dehydration.

### 4.2 Intraoperative care

#### 4.2.1 Prevention of intraoperative hypothermia

Maintaining normothermia is vital for ensuring normal metabolic processes. However, during surgery, factors such as anesthesia, a low ambient temperature in the operating room, and fluid administration may precipitate a drop in body temperature. Intraoperative hypothermia is associated with an increased risk of postoperative wound infections, impaired coagulation, and prolonged recovery times. During the postoperative recovery period, patients with low intraoperative temperatures often manifest with chills as the initial symptom, followed by arrhythmias and agitation. Based on robust evidence, the National Institute for Health and Care Excellence (NICE) recommends active prewarming and intraoperative warming measures for all adult surgical patients (43). Specific measures to maintain body temperature include:

Warming Devices: The judicious use of warming blankets or forced-air warming systems to maintain surface temperature and minimize heat loss.

Ambient Temperature Control: Adjusting the operating room temperature to between 22 °C and 24 °C.

Warmed Infusates: Employing fluid warmers for intravenous infusions or irrigation fluids.

Minimizing Exposure: Reducing the extent of patient exposure during surgery.

Thermal Conservation: Limiting heat loss associated with infusion fluids by using warmed physiological saline for wound irrigation (44–46).

#### 4.2.2 Optimization of anesthesia

Optimizing intraoperative anesthesia is a cornerstone of the ERAS protocol. Emphasis is placed on the precise titration of short-acting analgesics—such as propofol—to expedite postoperative recovery, paired with dynamic pain assessment systems that have been shown to reduce acute postoperative pain [with visual analog scale (VAS) scores decreasing by 30%–40%] (47, 48). Moreover, in adult laparoscopic procedures conforming to ERAS preoperative fasting guidelines, the use of laryngeal mask airway (LMA) ventilation—as opposed to conventional endotracheal intubation—has been demonstrated to significantly reduce the incidence of postoperative sore throat. LMA maintains efficient ventilation with stable oxygenation following extraperitoneal pneumoperitoneum (where CO_2_ gas is insufflated into the space posterior to the posterior rectus sheath and anterior to the peritoneum, achieving an intra-abdominal pressure of 11–13 mmHg), minimizes gastric distension, and offers a controllable aspiration risk, with outcomes comparable to those achieved via endotracheal intubation (49, 50).

#### 4.2.3 Catheter management

In adherence to the ERAS guideline of “minimizing invasive intervention,” our catheter management strategy avoids routine preoperative urinary catheterization to decrease patient discomfort (5). A urinary catheter is inserted only after induction of anesthesia if significant bladder distension impairs the surgical field. The duration of catheterization is strictly limited, with removal undertaken in the early phase of postoperative recovery. Studies indicate that early catheter removal does not significantly alter the incidence of postoperative urinary retention when compared to delayed removal. In fact, early removal is associated with reduced rates of catheter re-insertion, a lower incidence of catheter-associated urinary tract infections, diminished patient discomfort, and a shortened hospital stay (51, 52).

#### 4.2.4 Intraoperative pain management

Local infiltration anesthesia at the incision site—achieved via subcutaneous injection of 1% ropivacaine—effectively decreases the incidence of postoperative pain and reduces subsequent reliance on additional analgesics (53, 54). Recent clinical trials further demonstrate that the application of ropivacaine-soaked mesh at the wound site effectively mitigates acute pain intensity during the first 6 h postoperatively while streamlining multimodal analgesia management protocols (55).

### 4.3 Postoperative care

#### 4.3.1 Early mobilization

ERAS-oriented postoperative care strongly advocates for early mobilization to accelerate the recovery of bladder and gastrointestinal functions, decrease the incidence of urinary retention and abdominal distension, and lower the risk of deep vein thrombosis (36). Once patients regain sufficient consciousness, they are encouraged to engage in both passive and active limb exercises—such as turning, hip lifting, and joint flexion/extension. When postoperative electrocardiographic (ECG) monitoring reveals that vital signs have stabilized approximately 4 h after surgery and no other discomfort is present, monitoring can be discontinued to permit ambulation. Patients are then advised to walk slowly from the bedside, with prescribed walking distances marked in the ward corridors to facilitate gradual progression. This initiative has been met with high degrees of patient satisfaction.

#### 4.3.2 Postoperative pain management

Unbearable postoperative pain is one of the key factors that impede rapid patient recovery. Not only does severe pain reduce patient comfort, but it also interferes with crucial rehabilitation activities—such as early ambulation and timely resumption of oral intake—which can, in turn, delay the overall recovery process. The role of psychological factors in postoperative pain management should not be underestimated, as they play a critical role.

According to guidelines issued by the American Pain Society, ASRA, and ASA, the first step in a successful strategy is to collaborate with the patient in formulating a patient-centered, individualized pain management plan. This plan should encompass strategic preparation, goal setting, a detailed treatment regimen, and clear expectations regarding postoperative pain (56). Unfortunately, this valuable step is often overlooked during the initial preoperative consultation. Enhancing communication between healthcare providers and patients is essential—studies have shown that up to 94% of patients wish to be actively involved in decision-making, a factor that significantly boosts overall patient satisfaction (57).

Preoperatively, our registered nurses assess each patient's previous pain tolerance to establish a tailored analgesic regimen. Intraoperatively, the primary surgeon administers a 1% ropivacaine injection at the incision site to mitigate postoperative pain intensity. Postoperatively, our nursing staff conducts hourly pain assessments for the first 4 h, allowing timely adjustments to the management plan based on the patient's reported pain level. This protocol has consistently garnered high patient satisfaction and recognition.

Our postoperative pain management approach comprises the following two components:

Non-Pharmacological Pain Management:

Cognitive Behavioral Therapy (CBT):

By addressing and correcting catastrophic perceptions of pain, CBT helps reduce both anxiety and the intensity of pain. When applied in the perioperative setting—combining preoperative education with postoperative relaxation training—it can decrease the need for analgesics by 20%–30%.

Mindfulness-Based Stress Reduction (MBSR):

Under the guidance of our registered nurses, patients perform meditation and breathing exercises to regulate autonomic responses, which in turn helps lower cortisol levels associated with pain.

Biomechanical Therapy via Postural Optimization:

This technique involves selecting specific positions based on the pain site (for example, employing a semi-recumbent position at 30°–45° to reduce abdominal wall tension). Such positioning lowers the mechanical stress threshold of nociceptors, thereby contributing to effective pain relief.

Pharmacological Pain Management:

The first-line treatment is the administration of oral non-opioid analgesics (e.g., acetaminophen).

The alternative option is an intravenous injection of ketorolac tromethamine (58).

Importantly, opioid analgesics are strictly avoided to limit their use in the perioperative period. This multimodal approach not only promotes early ambulation but also encourages proactive nutritional improvement (59, 60).

#### 4.3.3 Postoperative dietary management

Our observations indicate that prolonged fasting and dehydration can exacerbate patient discomfort, heighten surgical anxiety, increase the likelihood of postoperative hypoglycemia and insulin resistance, disturb electrolyte balance, and intensify the stress response. To address these issues, we provide patients with specialized nutritional guidance and encourage the initiation of a moderate, high-protein diet by mouth approximately 4 h after surgery. This measure is designed to supply essential nutritional support, promote swift recovery, and mitigate the adverse effects associated with prolonged fasting and dehydration.

## 5 Challenges and future perspectives

SIL-TEP repair demands a high level of technical skill. Owing to the restricted operative space and frequent instrument clashes, the learning curve for SIL-TEP extends to approximately 45–60 cases, compared with only 25–30 cases for conventional TEP (29, 30). Under the framework of ERAS, although SIL-TEP presents inherent technical challenges and a shortage of long-term outcome data, its use has been associated with improved postoperative pain control, and superior cosmetic results—all without an added risk of complications. Future prospective randomized controlled trials (RCTs) are required to validate its long-term benefits, as well as to explore its potential integration with other innovative technologies such as artificial intelligence (AI) or robotic-assisted surgery. In the broader context of minimally invasive techniques, addressing disparities in medical resources and establishing personalized, precise treatment protocols remain critical challenges.

Looking forward, technological innovations hold great promise. The adoption of intelligent surgical systems and AI-based real-time navigation may help shorten the learning curve and further refine the ERAS pathway. Additionally, the integration of preoperative CT-based three-dimensional modeling of the hernia defect—for personalized mesh sizing—and the implementation of AI-powered early warning systems to predict complications, are expected to play pivotal roles in the evolution of surgical management.

## 6 Conclusion

Inguinal hernia is a common clinical condition whose treatment paradigm has shifted from traditional open surgery to minimally invasive laparoscopic techniques. Among these, SIL-TEP repair has emerged as a favored option due to its smaller incisions, enhanced cosmetic results, and accelerated postoperative recovery. Studies have demonstrated that, compared with conventional three-port TEP, SIL-TEP can reduce 24-h postoperative pain scores, shorten the time required for patients to resume daily activities, and elevate patient satisfaction regarding incision appearance. Nonetheless, the steep learning curve and technical challenges, such as instrument interference, remain significant obstacles.

The incorporation of ERAS protocols further optimizes the perioperative management of SIL-TEP. By promoting a multidisciplinary approach that encompasses preoperative dietary management, precision anesthesia during surgery, early postoperative oral intake, and multimodal analgesia, the ERAS pathway contributes to and a lower incidence of postoperative complications. Moreover, the intraoperative use of laryngeal mask airways has been shown to decrease the incidence of postoperative sore throat, and prompt removal of urinary catheters further minimizes the risk of urinary tract infections.

Despite these significant advantages, the combined application of SIL-TEP and ERAS faces challenges—particularly the limited evidence regarding long-term efficacy. Future advancements in technology and the further integration of precision medicine are anticipated to overcome these limitations.

Moving forward, efforts toward technical standardization, equitable resource allocation, and long-term follow-up studies are essential to ensure the widespread adoption and continuous refinement of this innovative approach.
